# Exogenous K^+^ enhances the desiccation tolerance and adhesion of *Pseudomonas protegens* SN15-2

**DOI:** 10.3389/fmicb.2026.1774231

**Published:** 2026-03-16

**Authors:** Jian Wang, Yaping Wang, Shufeng Bi, Wei Wang

**Affiliations:** 1 College of Life and Environmental Sciences, Huangshan University, Huangshan, China; 2 State Key Laboratory of Bioreactor Engineering, East China University of Science and Technology, Shanghai, China

**Keywords:** adhesion, desiccation stress, potassium, *Pseudomonas protegens*, survival

## Abstract

**Introduction:**

*Pseudomonas protegens* is an important plant growth-promoting rhizobacterium capable of both suppressing phytopathogens and enhancing plant growth. The ability of *P. protegens* to withstand desiccation stress is essential for its successful application in biocontrol.

**Methods:**

This study investigated the effects of potassium (K^+^) on the desiccation tolerance and adhesion ability of *P. protegens* through potassium supplementation during cultivation. In addition, transcriptome sequencing and gene overexpression analysis were used to investigate the mechanism by which K^+^ influences desiccation tolerance in *P. protegens*.

**Results:**

The addition of exogenous K^+^ was found to significantly enhance the survival of *P. protegens* under desiccation stress. Transcriptome analysis demonstrated that K^+^ induced the expression of multiple genes associated with nucleotide sugar biosynthesis and signal transduction, which are closely involved in bacterial stress tolerance. Additionally, K^+^ was observed to enhance the adhesion capability of *P. protegens*, thereby contributing to its successful colonization. Further experiments revealed that the GDP-mannose 4,6-dehydratase Gmd, whose expression is upregulated by K^+^, plays a pivotal role in the desiccation tolerance of *P. protegens*. The supplementation of K^+^ and the overexpression of Gmd were both found to markedly enhance the viability of *P. protegens* in microcapsule formulations under desiccation stress.

**Discussion:**

In summary, this study provides straightforward and effective protective strategies to improve the desiccation tolerance and biocontrol efficacy of *P. protegens*, which is of great significance for advancing its formulation and application.

## 1 Introduction

*Pseudomonas protegens*, a prominent plant growth-promoting rhizobacterium (PGPR) commonly found in various environmental habitats, exhibits a remarkable ability to suppress phytopathogens and enhance plant growth. Its biocontrol activity is largely attributed to the secretion of diverse antibiotics. For instance, 2, 4-diacetylphloroglucinol, a polyketide antibiotic synthesized by the *phl* gene cluster, is active against plant pathogenic fungi such as *Phytophthora ramorum* and *Botrytis cinerea* (Balthazar et al., 2022). The aromatic polyketide antibiotic pyoluteorin, synthesized by the *plt* gene cluster, significantly inhibits the growth of *Pythium* spp. and *B. cinerea* (Shi et al., 2019). Phenazine-1-carboxylic acid, synthesized by the *phz* gene cluster, inhibits the growth of *Xanthomonas oryzae* by disrupting its cellular redox balance (Xu et al., 2015). Beyond pathogen suppression, *P. protegens* can also promotes plant growth and enhance plant resilience against abiotic stresses through mechanisms such as the secretion of indole-3-acetic acid (IAA) and ACC deaminase (Glick and Nascimento, 2021; Mehmood et al., 2023). Inoculation with *P. protegens* DA1.2 has been shown to mitigate drought stress and promote growth in wheat (Bakaeva et al., 2022). These multifaceted beneficial traits make *P. protegens* a highly promising candidate for development as a commercial biocontrol agent.

However, the successful commercialization and field efficacy of such biocontrol agents are critically limited by their ability to withstand harsh processing and environmental conditions. During the production of formulated inoculants, microbial cells are subjected to desiccation stress, which drastically reduces the viability of non-sporulating bacteria like *P. protegens* (Stephan et al., 2016). Furthermore, in field applications, the bacterium often faces environmental stresses such as desiccation and UV radiation, which can critically undermine their survival and colonization—the prerequisite for exerting their beneficial functions (Nguyen et al., 2025). Therefore, enhancing the desiccation tolerance and ecological fitness of *P. protegens* is crucial for its successful commercialization and application.

A common approach to improving microbial stress tolerance involves the incorporation of protective additives, such as sugars and osmolytes, into cultivation and formulation matrices. The cell viability of *Pseudomonas fluorescens* EPS62e after freeze-drying was enhanced by using lactose as a protectant (Cabrefiga et al., 2014). Isomelezitose has been successfully applied as a protectant for *P. fluorescens* biocontrol agent during drying and storage (Slininger et al., 2021). Crucially, the protective efficacy of any given additive is highly strain-specific (Zhan et al., 2012). Several studies indicate that intracellular osmoprotectant concentration is a key determinant of bacterial survival during desiccation. The accumulation of trehalose has been shown to confer protection to *Lactococcus lactis* during the process of freeze-drying (Termont et al., 2006). While the effect of glycine betaine accumulation is highly strain-dependent, exerting either positive or negative impacts on desiccation tolerance (Sheehan et al., 2006; Bergenholtz et al., 2012). As the predominant intracellular cation, potassium (K^+^) has a central function in key bacterial processes such as osmoadaptation, gene expression, and enzyme activation (Epstein, 2003). Our previous research identified K^+^ as an osmoprotectant that enhances hyperosmotic stress tolerance in *P. protegens* (Wang et al., 2024b). However, the potential of K^+^ to enhance the bacterium’s desiccation tolerance remained to be elucidated.

Therefore, this study was designed to systematically investigate the effect and mechanism of K^+^ on the desiccation tolerance of *P. protegens* SN15-2, a strain with excellent biocontrol ability against plant pathogen *Ralstonia solanacearum* (Lou et al., 2018). We confirmed that K^+^ significantly improve the survival rate of *P. protegens* after desiccation. Exogenous K^+^ also improved bacterial adhesion capacity. Transcriptomic analysis revealed that potassium induction upregulates several genes involved in polysaccharide biosynthesis, adhesion, and signal transduction. Among these upregulated genes, overexpression of *gmd*, which encodes GDP-mannose 4,6-dehydratase, significantly enhanced the desiccation tolerance of *P. protegens*. In summary, these findings provide deeper insights into strategies for improving the desiccation tolerance and efficacy of *P. protegens* as a biocontrol agent.

## 2 Materials and methods

### 2.1 Bacterial strains and culture conditions

Details of the bacterial strains and plasmids are provided in Table 1. *Escherichia coli* strains were cultivated in LB medium at 37 °C. *Pseudomonas protegens* strains were grown in NB medium at 30 °C. The formulation of the culture medium is detailed in the previous study (Wang et al., 2024b). Where required, KCl was supplemented to the NB medium at final concentrations of 0, 0.5, 1, 5, 10, or 20 mM. Gentamicin was added to the medium at a concentration of 50 μg/mL when necessary.

**TABLE 1:** Strains and plasmids used in this study.

Strain or plasmid	Description	References
**Strains**		
*P. protegens* SN15-2	Wild type, soil isolate, CGMCC No: 17211	Lou et al., 2018
WT-EGFP	*P. protegens* SN15-2 harboring pMP2444, Gm*^r^*	This study
WT-*gmd*	*P. protegens* SN15-2 harboring overexpressed plasmid pBBR2-*gmd*, Gm*^r^*	This study
WT-pBBR	*P. protegens* SN15-2 harboring plasmid pBBR1MCS-2, Gm*^r^*	This study
*E. coli* DH5α	λ^–^ϕ80d*lacZ*Δ*M15*Δ(*lacZYA-argF*)*U169 recA1 endA1 hsdR17*(r_K_^–^ m_K_^–^) *supE44 thi-1 gyrA relA1*	Lab collection
**Plasmids**		
pMP2444	Plasmid broad host range Gm^r^, containing *egfp*, Gm^r^	Ramirez-Mata et al., 2018
pBBR1MCS-2	Mobilisable shuttle and expression vector; Gm^r^	Kovach et al., 1995
pBBR-*gmd*	pBBR1MCS-2 containing the intact *gmd* gene; Gm^r^	This study

Gm^r^ indicates gentamicin resistance.

### 2.2 Growth and desiccation assays

*P. protegens* SN15-2 and its derivatives were grown overnight and then normalized to an OD600 of 0.8. The cell suspensions were then diluted 1:100 in NB medium supplemented with varying concentrations of KCl (0, 0.5, 1, 5, 10, and 20 mM). A control containing 10 mM NaCl was incorporated to account for any potential effects attributable to chloride. The cultures were shaken at 200 rpm at 30 °C. After 20 h of incubation, the cells were harvested and subjected to the desiccation tolerance assay.

For the desiccation assay, 10 μL aliquots of bacterial culture were spotted onto mixed cellulose ester membranes (0.22 μm pore size) placed in sterile Petri dishes, following a previously described method (Velázquez-Hernández et al., 2011). The plates were then placed in a sealed desiccator containing a saturated NaCl solution to maintain a relative humidity of approximately 75% at 25 °C (Stockwell et al., 2009). After 120 min of desiccation, bacterial cells were retrieved from the membranes by vortexing in sterile saline for 10 min. Subsequent serial dilutions of the resulting suspensions were plated to determine viable counts. The survival rate was calculated as the percentage of colony-forming units (CFU) in the desiccated samples relative to the non-desiccated control.

### 2.3 RNA sequencing analysis

*P. protegens* SN15-2 was cultivated in NB medium at 30 °C, containing either 0 mM or 10 mM KCl, to reach the stationary phase. The extraction of RNA was performed on cells obtained from triplicate biological samples for each condition. RNA quality was verified using an Agilent 2100 Bioanalyzer, and concentration was measured with a NanoDrop ND-2000. Subsequent library preparation involved ribosomal RNA removal, mRNA fragmentation, and double-stranded cDNA synthesis using a SuperScript kit (Invitrogen). Sequencing was conducted on an Illumina HiSeq 4000 platform. Transcript levels were quantified through the RPKM method (Mortazavi et al., 2008). Differentially expressed genes (DEGs) were identified using DESeq2, with thresholds set at a fold change > 2 and an FDR-adjusted *p*-value < 0.05 (Love et al., 2014). The raw RNA sequencing data have been deposited in the NCBI Sequence Read Archive (SRA) under BioProject accession number PRJNA1391811.

### 2.4 Adhesion assays

Bacterial adhesion under static conditions was evaluated using a 24-well plate assay as described (Rima et al., 2025). Briefly, *P. protegens* were incubated in fresh NB medium with or without the addition of 10 mM K^+^ at 30 °C for 20 h. The cultures were then transferred to a 24-well plate (Falcon, TC-treated, polystyrene) and maintained statically at 30 °C for 4 h. After incubation, gentle washing was performed to remove non-adherent cells. The adherent biomass was fixed and stained with 0.1% crystal violet. The dye associated with the adhered cells was subsequently solubilized with 95% ethanol, and its absorbance was measured at 595 nm to quantify bacterial adhesion.

For fluorescence-based visualization, the plasmid pMP2444 (Ramirez-Mata et al., 2018), which constitutively expresses enhanced green fluorescent protein (EGFP), was introduced into wild-type *P. protegens* SN15-2 via electroporation, following an established protocol (Wang et al., 2024a). Overnight cultures of the green fluorescent tagged strain WT-EGFP were spotted onto the center of sterile microscope slides. The slides were maintained in a humid chamber at 30 °C for 4 h to allow cell attachment. Subsequently, loosely attached cells were removed by gently rinsing the slides twice in 100 mL of sterile water. Bacterial adhesion and microcolony formation on the glass surface were then visualized using fluorescence microscope.

### 2.5 Tomato leaves colonization assays

*P. protegens* SN15-2 was grown to stationary phase in NB medium containing 0 mM or 10 mM KCl. Cells were harvested by centrifugation and normalized to 1 × 10^9^CFU/mL in sterile water. The inoculum was conducted as previously described (Bonaterra et al., 2007). Tomato plants (six weeks old) were sprayed with the bacterial suspension until runoff (approximately 10 mL per plant). After inoculation, the plants were cultivated in chambers under controlled conditions (30 °C, 50% RH, 16-h light/8-h dark photoperiod). The population dynamics of *P. protegens* on tomato plants were monitored by collecting leaf samples at 1 and 7 days post-inoculation. Four leaves were randomly sampled from each replicate plant. To quantify epiphytic colonization, sampled leaves were vortexed in sterile water for 30 min to dislodge surface-associated bacteria. The resulting suspension was then serially diluted and plated for colony counting. To quantify endophytic colonization, leaves were first surface-sterilized to eliminate epiphytic bacteria, then homogenized in sterile water. The homogenate was serially diluted and plated. For both assays, the population density was calculated and expressed as colony-forming units per gram of fresh leaf weight.

### 2.6 Construction of *gmd* overexpression strain

The gmd gene overexpression strain was constructed following established protocols (Kovach et al., 1995; Wu et al., 2021). The shuttle plasmid pBBR1MCS-2 was digested with restriction endonuclease EcoRI and HindIII. The gmd coding sequence was amplified by PCR using wild-type *P. protegens* SN15-2 genomic DNA as the template. The purified PCR product was then ligated into the linearized pBBR1MCS-2 vector using an Uniclone One Step Seamless Cloning Kit (Genesand) to obtain the recombinant overexpression plasmid pBBR-*gmd*. Both the recombinant plasmid pBBR-*gmd* and the empty vector pBBR1MCS-2 were subsequently electroporated into *P. protegens* SN15-2 to generate the overexpression strain WT-*gmd* and the empty vector control strain WT-pBBR, respectively. All constructed strains were confirmed by PCR amplification and Sanger sequencing. The oligonucleotide sequences used are provided in Supplementary Table 1.

### 2.7 Evaluation of *P. protegens* survival in microcapsules

*P. protegens* SN15-2 was grown to the stationary phase in NB medium at 30°C, supplemented with either 0 mM or 10 mM KCl. Cells were harvested by centrifugation and subsequently washed with sterile water to eliminate residual nutrients carried from the NB medium. Bacterial encapsulation was performed under sterile conditions using an extrusion technique (He et al., 2016; Pour et al., 2019). Briefly, a sterile composite solution was prepared with sodium alginate (NaAlg) and starch at final concentrations of 2% (w/v) and 3% (w/v), respectively. This solution was then mixed evenly with the *P. protegens* cell suspension (5 × 10^9^ CFU/ml) at a 2:1 (v/v) ratio. The mixture was extruded via a 0.9 mm injection needle and added dropwise into a 2% (w/v) CaCl_2_ crosslinking solution. After 1 h of bead formation, the microcapsules were washed three times in sterile water. The wet beads were collected and dried in an air-drying oven at 30 °C for 6 h to constant weight. The dried biocontrol formulations were hermetically sealed and maintained at 4 °C. The viability of encapsulated bacteria was quantified immediately after drying and after the 30-day storage period. For each assay, 1 g of dried beads was aseptically rehydrated in 10 mL sterile saline for 2 h at room temperature. The swollen beads were then homogenized to release the encapsulated bacteria, and viable counts were obtained via the standard spread plate method.

### 2.8 Quantitative real-time PCR (qRT-PCR)

Following total RNA extraction with TRIzol Reagent (Invitrogen), cDNA was reverse transcribed using StarScript II RT Mix (GenStar). Quantitative PCR was conducted on a BioRad CFX96 employing 2 × RealStar Power SYBR qPCR Mix (GenStar). The 16S rRNA gene served as the endogenous reference for normalization, and gene-specific primers are listed in Supplementary Table 1. Relative expression levels were calculated using the 2^−ΔΔ^*^Ct^* method (Schmittgen and Livak, 2008).

### 2.9 Statistical analysis

All experiments were performed in triplicate and repeated three times. Data are presented as the mean ± standard deviation (SD). Statistical significance was determined using Student’s *t*-test, with a threshold of *p*-value < 0.05.

## 3 Results

### 3.1 K^+^ enhances the desiccation tolerance of *Pseudomonas protegens* SN15-2

**FIGURE 1:**
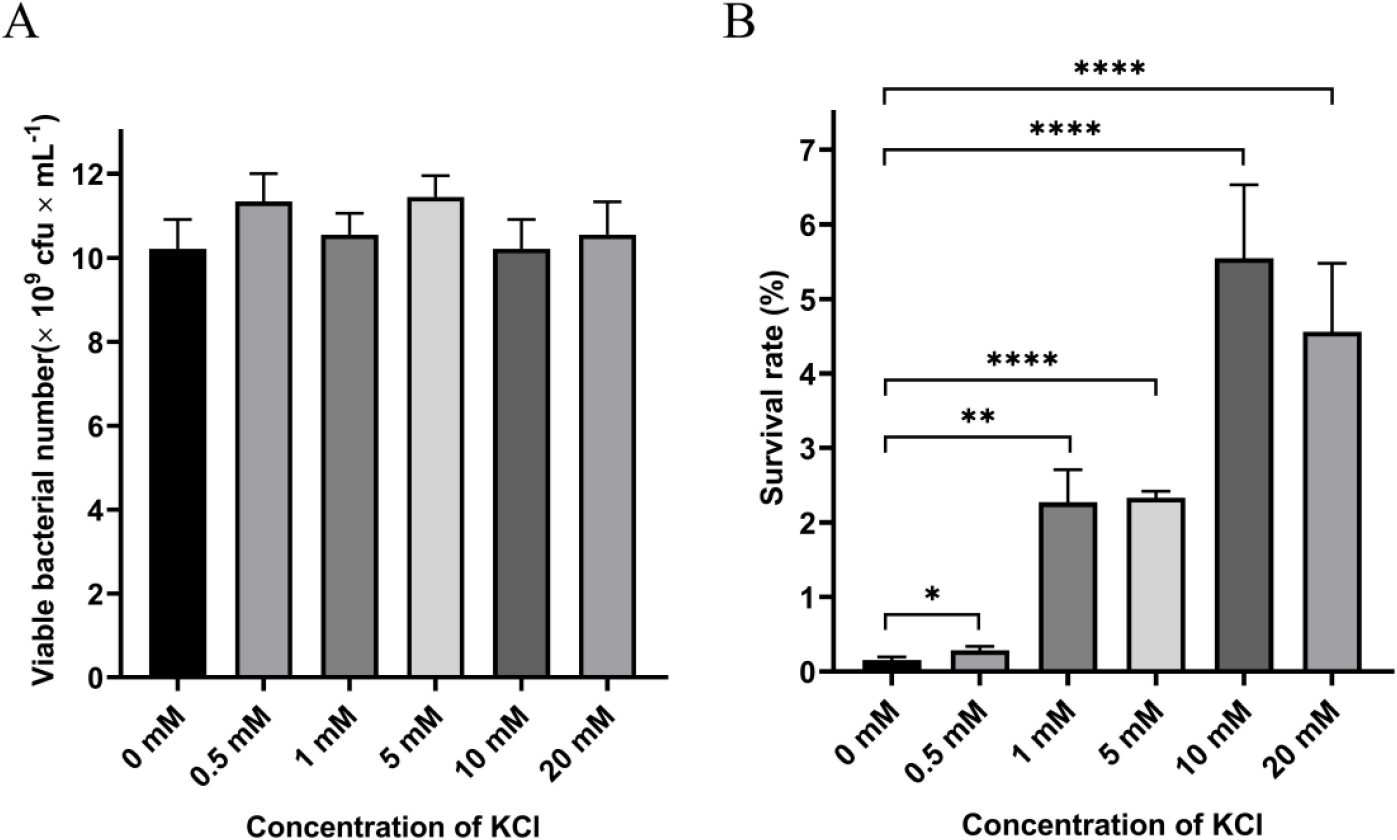
Effect of exogenous potassium ion (K^+^) on the growth **(A)** and desiccation tolerance **(B)** of *P. protegens* SN15-2. Error bars represent the standard deviations. Statistical significance was determined using Student’s *t*-tests. *P < 0.05; **P < 0.01; ****P < 0.0001.

*P. protegens* SN15-2 was grown in NB medium supplemented with KCl at concentrations ranging from 0 to 20 mM. The results revealed that exogenous K^+^ had no significant influence on the growth of *P. protegens* (Figure 1A). In contrast, KCl notably improved the survival rate of *P. protegens* following desiccation stress in a concentration-dependent manner. The optimal survival rate, observed at 10 mM KCl, was 36.25 times greater than that of the control lacking K^+^ (Figure 1B). Importantly, a 10 mM NaCl control showed no effect on post-desiccation survival, ruling out a contribution from chloride ions (Supplementary Figure 1). These findings clearly demonstrate that exogenous K^+^ plays an important role in enhancing the desiccation tolerance of *P. protegens*.

### 3.2 Analysis of potassium-induced differential gene expression in *P. protegens* SN15-2

**FIGURE 2:**
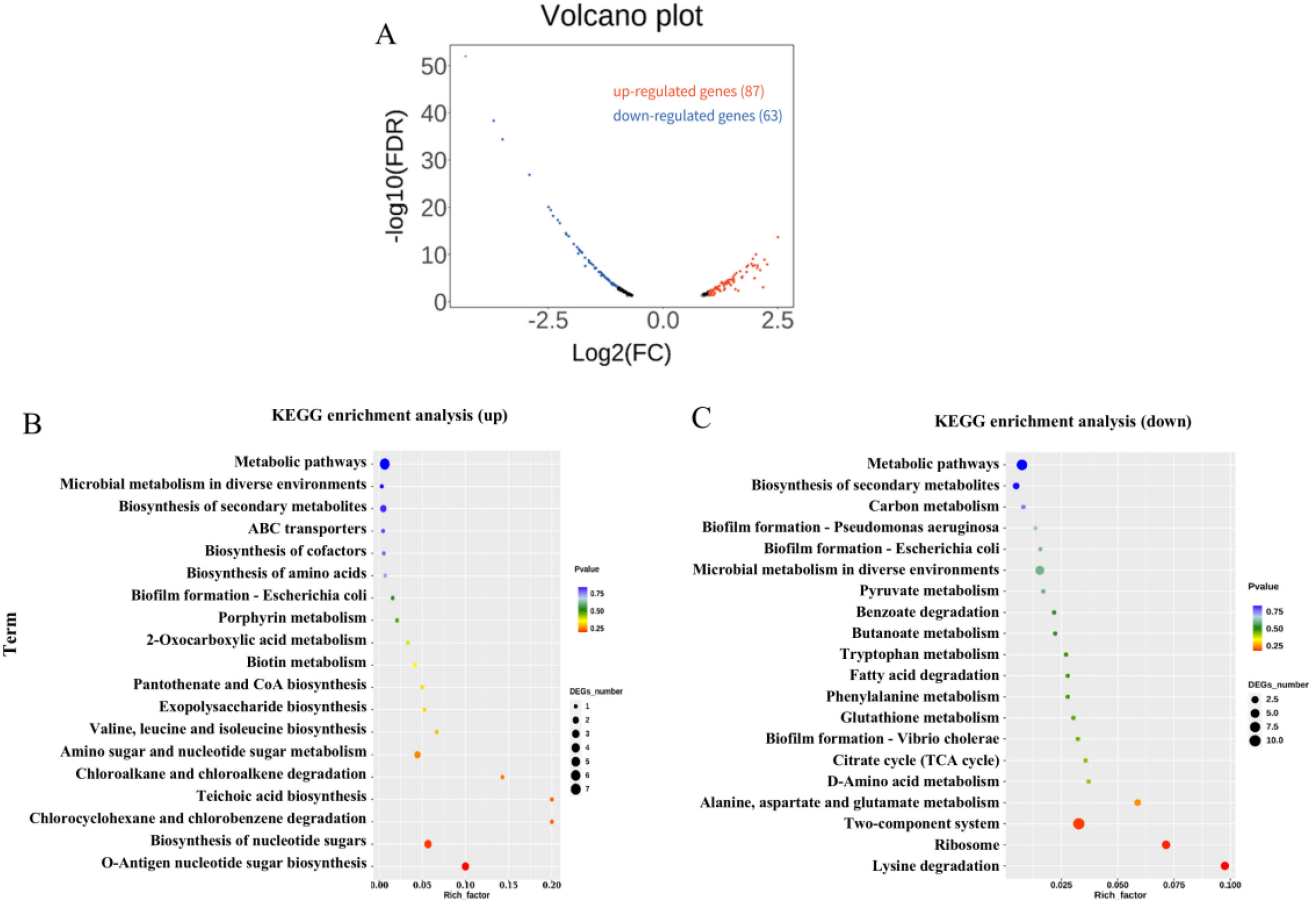
Comparative transcriptome profiling of *P. protegens* SN15-2 with and without K^+^. **(A)** Volcano plot of differentially expressed genes (DEGs) affected by K^+^, with up- and down-regulated genes highlighted in red and blue, respectively. **(B)** KEGG pathway enrichment analysis of upregulated genes. **(C)** KEGG pathway enrichment analysis of downregulated genes.

To elucidate the mechanism underlying K^+^-enhanced desiccation tolerance in *P. protegens*, transcriptomic profiles were compared between cells cultivated in NB medium supplemented with or without 10 mM K^+^. Comparative transcriptome analysis identified 150 differentially expressed genes (DEGs), of which 87 were upregulated and 63 were downregulated (Figure 2A). The qRT-PCR confirmed the expression levels of 10 DEGs, chosen based on statistical significance and functional relevance to polysaccharides biosynthesis (Supplementary Figure 2). KEGG pathway enrichment analysis revealed that upregulated genes were significantly enriched in O-antigen nucleotide sugar biosynthesis and nucleotide sugar biosynthesis pathways (Figure 2B). Meanwhile, downregulated genes were primarily enriched in lysine degradation, ribosome biogenesis, and two-component system pathways (Figure 2C). These findings indicate that 10 mM exogenous K^+^ modulates multiple cellular functions in *P. protegens* SN15-2, which may underlie its enhanced desiccation stress tolerance.

### 3.3 K^+^ enhances the adhesive capacity of *P. protegens* SN15-2

**FIGURE 3:**
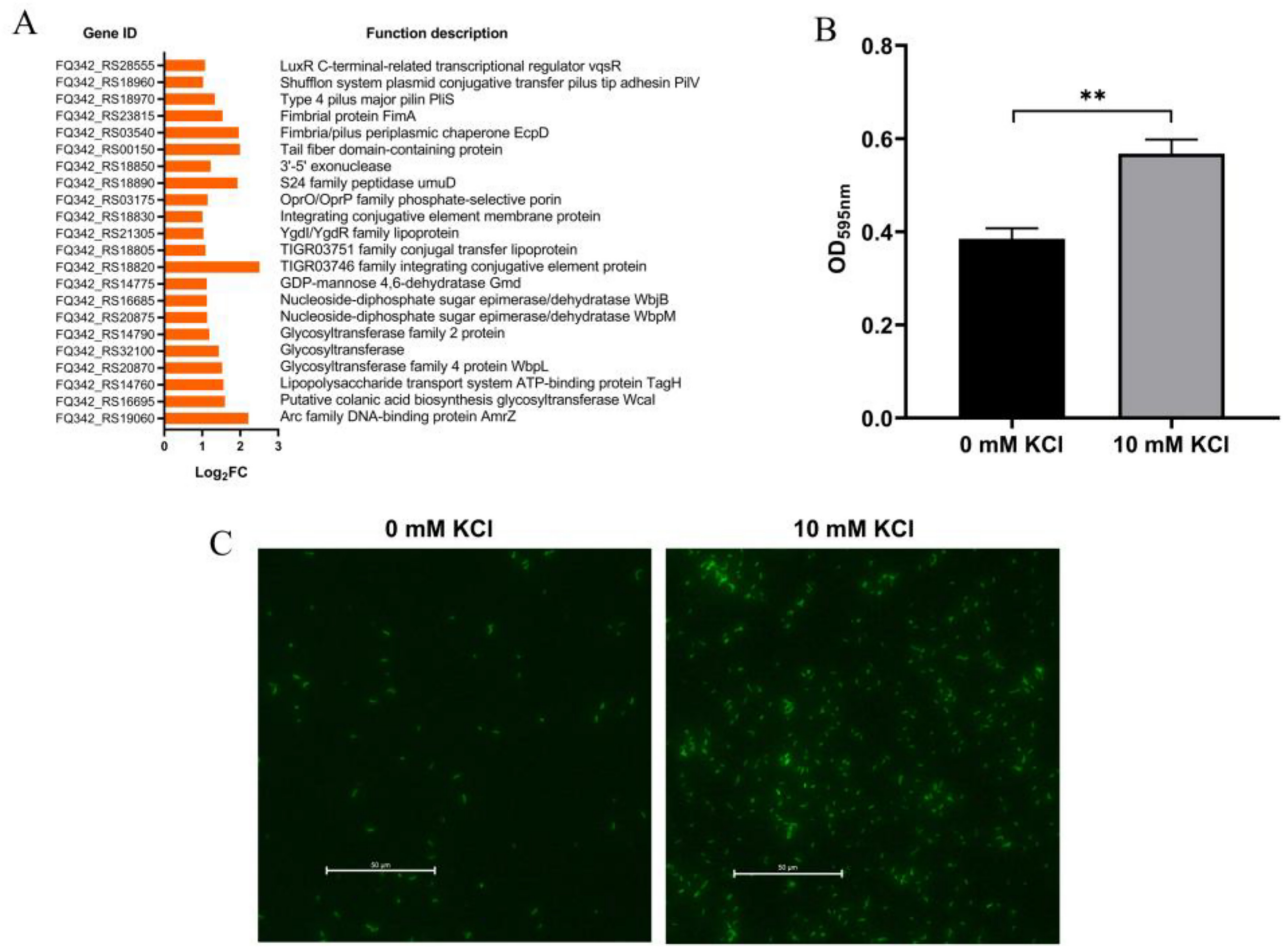
Effect of exogenous K^+^ on the adhesion ability of *P. protegens* SN15-2. **(A)** Transcriptomic analysis reveals K^+^-induced upregulation of genes associated with adhesion and stress tolerance. **(B)** Quantitative crystal violet staining shows enhanced *P. protegens* adhesion to polystyrene surfaces with exogenous K^+^. **(C)** Fluorescence microscopy of EGFP-tagged cells confirms stronger adhesion to glass surfaces in the K^+^-treated group. Error bars represent the standard deviations. Statistical significance was determined using Student’s *t*-tests. **P < 0.01.

Adhesion capability is intimately involved in the survival and successful colonization of plant growth-promoting rhizobacteria (Costa-Gutierrez et al., 2022; Huang et al., 2022). Transcriptomic analysis revealed that K^+^ significantly upregulated genes related to pilus assembly, glycosyltransferases activity, and the biosynthesis of lipopolysaccharides and capsular polysaccharides (Figure 3A). Functionally, these genes are associated with adhesion and desiccation stress tolerance—a connection that will be explored in detail in the Discussion. Consistent with the transcriptome data, crystal violet staining assays demonstrated that K^+^ markedly enhanced the adhesion of *P. protegens* to polystyrene surfaces (Figure 3B). To directly visualize bacterial adhesion, we introduced the enhanced green fluorescent protein (EGFP)-expressing plasmid, pMP2444, into wide-type *P. protegens* SN15-2. A markedly stronger fluorescent signal was observed via fluorescence microscopy in the K^+^-treated group, providing clear evidence for enhanced bacterial adhesion on the glass surface (Figure 3C). Together, these results collectively demonstrate that K^+^ significantly enhance the adhesive capacity of *P. protegens* SN15-2.

### 3.4 K^+^ enhances the colonization of *P. protegens* SN15-2 on tomato leaves

**FIGURE 4:**
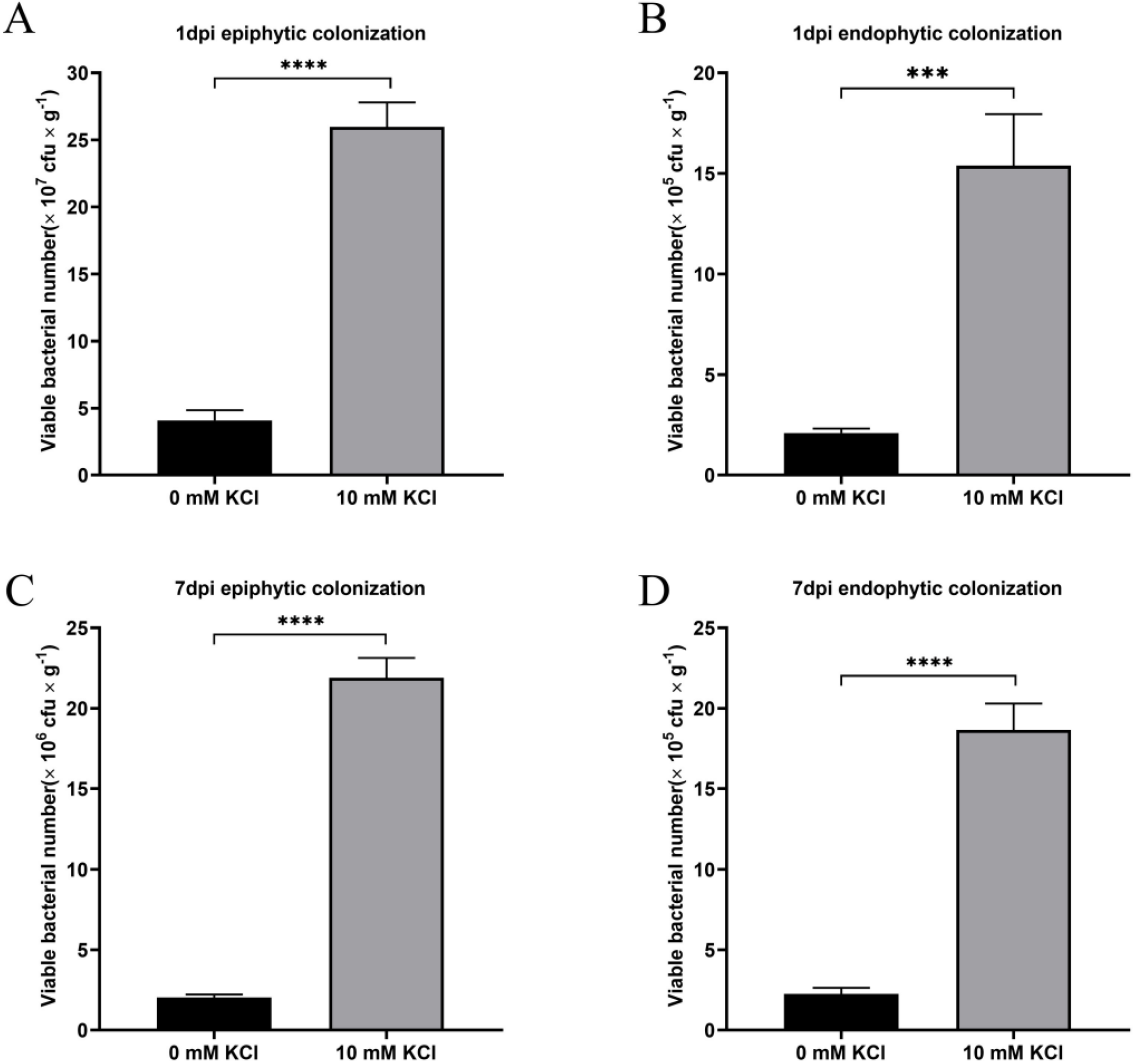
Effects of K^+^ on the colonization ability of *P. protegens* on tomato leaves. *P. protegens* epiphytic colonization **(A)** and endophytic colonization **(B)** at 1 day post-inoculation (dpi). *P. protegens* epiphytic colonization **(C)** and endophytic colonization **(D)** at 7dpi. Data are shown as colony-forming units (CFU) per gram of fresh leaf weight. Error bars represent the standard deviations. Statistical significance was determined using Student’s *t*-tests. ***P < 0.001; ****P < 0.0001.

Tomato seedlings were spray-inoculated with *P. protegens*, and the epiphytic and endophytic populations of *P. protegens* on leaves were periodically monitored. Our results demonstrated that exogenous K^+^ significantly enhanced the colonization of *P. protegens* in both the epiphytic and endophytic niches of tomato leaves. At 1 day post-inoculation (dpi), the epiphytic population of the K^+^-supplemented group reached a level of 25.97 × 10^7^ CFU/g fresh leaf weight, which was 6.36-fold greater than that of the non-K^+^-supplemented control group (Figure 4A). At 1 dpi, the endophytic colonization population of the K^+^-supplemented group reached 15.38 × 10^5^ CFU/g fresh leaf weight, which was 7.35-fold higher than the value observed in the control group without K^+^ addition (Figure 4B). At 7 dpi, the K^+^-supplemented group supported a significantly greater epiphytic bacterial population than the control, achieving 21.89 × 10^6^ CFU/g fresh leaf weight (Figure 4C). The K^+^-supplemented group also showed a significant increase in endophytic colonization relative to the control at 7 dpi (Figure 4D). Collectively, these findings indicate that exogenous K^+^ can markedly improve the colonization capacity of *P. protegens* on tomato plants, which is closely associated with the enhanced desiccation tolerance and adhesion ability of *P. protegens* induced by K^+^.

### 3.5 Overexpression of *gmd* enhances desiccation tolerance of *P. protegens*

**FIGURE 5:**
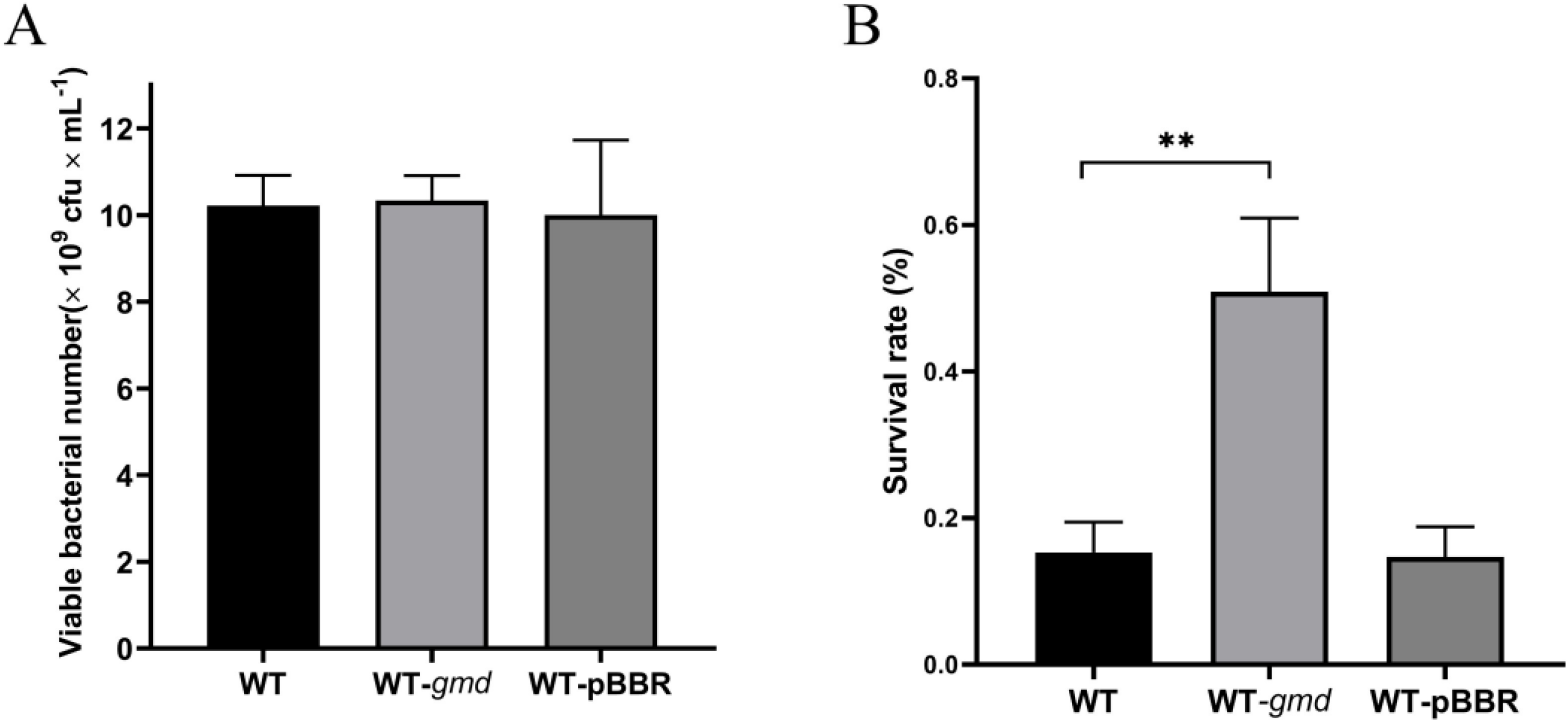
Role of GDP-mannose 4,6-dehydratase Gmd in desiccation tolerance of *P. protegens*. **(A)** Effect of *gmd* overexpression on bacterial growth. **(B)** Effect of *gmd* overexpression on desiccation tolerance. Error bars represent the standard deviations. Statistical significance was determined using Student’s *t*-tests. **P < 0.01.

The expression of *gmd*, which encodes GDP-mannose 4,6-dehydratase Gmd, was significantly upregulated under K^+^ induction (Figure 3A). Gmd catalyzes a key step in cell-surface polysaccharide biosynthesis, which is closely linked to bacterial environmental stress tolerance (King et al., 2009). Therefore, we constructed a *gmd* overexpression strain using the pBBR1MCS-2 vector to assess its effect on the desiccation tolerance of *P. protegens*. While *gmd* overexpression had no significantly impact on bacterial growth, it markedly increased the survival of *P. protegens* following desiccation stress (Figure 5). Collectively, these results highlight that *gmd*, whose expression is upregulated by K^+^, plays a critical role in enhancing the desiccation tolerance of *P. protegens*.

### 3.6 K^+^ promotes survival of *P. protegens* SN15-2 in microcapsules

**FIGURE 6:**
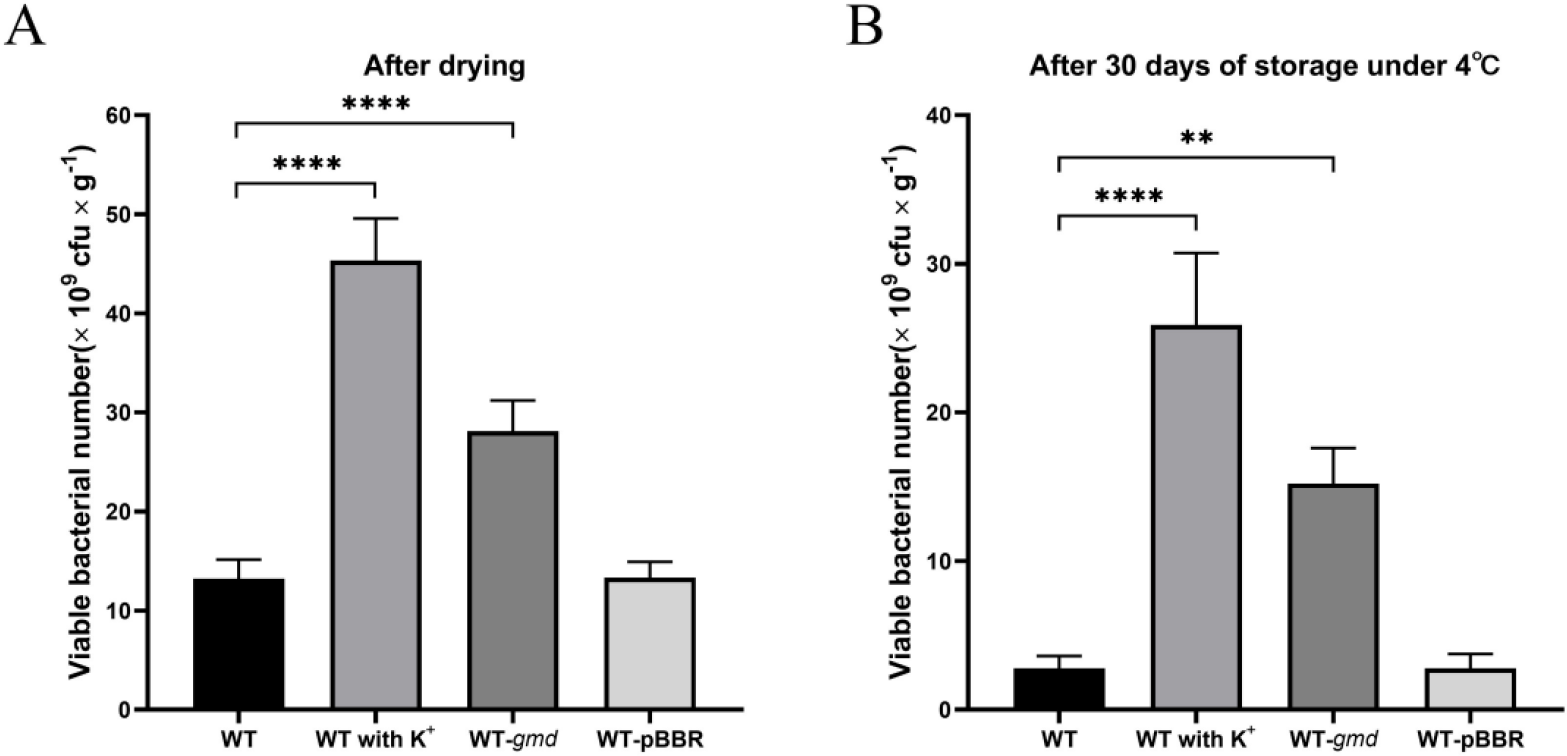
Effects of exogenous K^+^ and *gmd* overexpression on survival of *P. protegens* in microcapsule. **(A)** Survival rate immediately after drying. **(B)** Survival rate after 30 days of storage at 4 °C. Error bars represent the standard deviations. Statistical significance was determined using Student’s *t*-tests. **P < 0.01; ****P < 0.0001.

To assess the impact of potassium ions on the viability of *P. protegens* in sodium alginate microcapsules, we quantified the viable cell counts immediately after air-drying and following 30 days of storage at 4 °C. The results indicated that exogenous K^+^ significantly enhanced bacterial survival. Immediately after drying, the number of viable bacteria in the K^+^-supplemented formulation was 3.43 times higher than in the non-supplemented control (Figure 6A), consistent with the role of K^+^ in enhancing cellular desiccation tolerance. After 30 days of storage, the K^+^-treated group maintained a 9.32-fold higher viable count than the control (Figure 6B), demonstrating that K^+^ significantly improves formulation stability. Similarly, the *gmd*-overexpressing strain exhibited superior survival. Its viability was 2.13 times greater than that of the wild-type strain immediately after drying (Figure 6A), and this 5.48-fold advantage persisted after 30 days of storage (Figure 6B). These results indicate that both exogenous K^+^ and *gmd* overexpression substantially enhance the long-term survival of *P. protegens* in dried formulations.

## 4 Discussion

Desiccation, which leads to severe cellular dehydration, represents one of the most prevalent and critical stresses encountered by bacteria in natural environments. *Pseudomonas protegens* is a highly promising candidate for commercial development as a biocontrol agent due to its capacity to antagonize plant pathogens and stimulate plant growth. However, as a non-sporulating bacterium, *P. protegens* is inherently susceptible to desiccation stress, a key limitation for its large-scale formulation and application. Common strategies to improve microbial desiccation tolerance include stress pre-adaptation and the supplementation of protective compounds. The cell viability of *Pseudomonas fluorescens* EPS62e after freeze-drying was improved by cultivation under osmoadaptive conditions combined with the use of lactose as a protectant (Cabrefiga et al., 2014). Preadaptation with 0.05 wt% H_2_O_2_ for 30 min significantly improved the survival of *P. fluorescens* after cold-air drying during biocontrol agent preparation (Wu et al., 2021). The addition of exogenous trehalose coupled with overexpression of a trehalose transporter markedly improved the desiccation tolerance of *Saccharomyces cerevisiae* (Tapia et al., 2015). This study provides the first demonstration that K^+^ supplementation during cultivation markedly increases the desiccation tolerance of *P. protegens* and offers preliminary insights into the protective mechanisms involved.

K^+^ are integral to fundamental cellular processes in microorganisms. In most halophiles, K^+^ serves as a primary compatible solute, playing a critical role in maintaining osmotic homeostasis (Meng et al., 2019). Our previous work established that exogenous K^+^ significantly improves the growth of *P. protegens* SN15-2 under hyperosmotic stress (Wang et al., 2024b). While osmotic stress differs from desiccation stress, both conditions challenge bacterial cells by reducing water activity. Given this shared physiological challenge and the established function of K^+^ as an effective osmoprotectant, this study was designed to systematically evaluate its potential to enhance bacterial survival under desiccation conditions. A key finding of this study is that exogenous K^+^ confers a significant increase in the desiccation tolerance of *P. protegens*. This represents the first documented evidence of such a protective role for K^+^ in *P. protegens*, an advance that holds significant promise for improving the viability and commercial utility of *P. protegens*-based biocontrol formulations.

To elucidate the molecular mechanism underlying K^+^-enhanced desiccation tolerance, we performed transcriptome sequencing of *P. protegens*. The analysis revealed a significant upregulation of genes encoding key regulatory proteins, including the quorum-sensing regulator VqsR and the c-di-GMP signaling regulator AmrZ (Li et al., 2007; Muriel et al., 2018). Quorum sensing and c-di-GMP-mediated signaling play pivotal roles in governing environmental stress adaptation in pseudomonads (Tang et al., 2019; Huo et al., 2026). Additionally, the expression of UmuD, which is involved in DNA damage response (Sutton et al., 2002), was significantly upregulated in response to K^+^. Given that desiccation inflicts substantial DNA damage (Greffe and Michiels, 2020), the upregulation of this DNA repair protein likely constitutes an important component of the enhanced tolerance mechanism. Collectively, these transcriptional changes suggest that exogenous K^+^ activates a coordinated defense network in *P. protegens*, involving stress-signaling pathways and DNA repair systems, to mitigate the effects of desiccation.

The outermost protective barrier of Gram-negative bacteria is the outer membrane containing lipopolysaccharide (LPS). Structurally intact LPS plays a critical role in protecting cells against desiccation, as demonstrated in *Rhizobium leguminosarum*, where LPS contributes to both desiccation tolerance and osmotic stress tolerance (Vanderlinde et al., 2009). *Acinetobacter baumannii* enhances desiccation resistance by modifying its lipid A structure to strengthen the outer membrane (Boll et al., 2015). Conversely, the absence of LPS significantly impairs the desiccation tolerance of *Salmonella enterica* (Garmiri et al., 2008). An additional key extracellular protective layer is the capsule exopolysaccharide (CPS), known to confer desiccation tolerance in several bacterial species. *Staphylococcus aureus* increases its desiccation tolerance under prolonged dry conditions by enhancing CPS production (Wang et al., 2022). Correspondingly, CPS production deficiency reduces the survival rates under desiccation conditions of *Elizabethkingia miricola* (Hu et al., 2024). Together, LPS and CPS constitute a synergistic barrier that protects Gram-negative bacteria against adverse environmental conditions. The O-antigen, a component of LPS located at the outermost part of the molecule, plays a important role in this protection. The O-antigen is essential for *Salmonella enterica* to survive desiccation stress (Gibson et al., 2006). Nucleotide sugars serve as the fundamental building blocks for cell surface-associated polysaccharide. In this study, transcriptome analysis revealed that genes upregulated in response to K^+^ were significantly enriched in the O-antigen nucleotide sugar biosynthesis and biosynthesis of nucleotide sugars pathways. These findings indicate that K^+^ may enhance the desiccation tolerance of *P. protegens* by upregulating the synthesis of both LPS and CPS.

In *P. aeruginosa*, GDP-mannose 4,6-dehydratase (Gmd) catalyzes the conversion of GDP-D-mannose to GDP-4-keto-6-deoxy-D-mannose (Mäki et al., 2002). This key intermediate can then be reduced to the GDP-monodeoxyhexose, GDP-l-fucose, GDP-d-rhamnose, GDP-deoxy-d-talose or GDP-deoxy-d-altrose. Bacteria utilize these sugars to synthesize surface-associated polysaccharides, which form protective barriers that enhance bacterial stability and adaptability under adverse conditions. Given the pivotal role of Gmd in polysaccharide biosynthesis, we investigated its contribution to desiccation tolerance in *P. protegens*. A *gmd*-overexpressing mutant was successfully constructed and showed a markedly increased survival rate following desiccation relative to the wild-type *P. protegens*. This finding demonstrates that Gmd contributes to the enhancement of desiccation tolerance in *P. protegens*, highlighting its potential as a genetic target for improving the strain’s ability to withstand desiccation conditions.

Bacterial surface polysaccharides, such as lipopolysaccharides, capsular polysaccharides, and exopolysaccharides, function not only as protective barriers but also play critical roles in surface adhesion (Patro and Rathinavelan, 2019). Pilus is also a major contributor to the bacteria’s adhesion ability (Ochner et al., 2024). Transcriptome data revealed that K^+^ not only induced the upregulation of genes associated with cell-surface polysaccharide synthesis but also promoted the expression of several pilus-related genes, including *pliV*, *pliS*, and *ecpD*. We therefore hypothesized that the adhesive capacity of *P. protegens* is enhanced by exogenous K^+^. This hypothesis was confirmed by crystal violet staining and fluorescence-based adhesion assays, which demonstrated that K^+^ significantly increased bacterial adhesion to solid surfaces. For biocontrol agents, adhesion capability is closely linked to colonization efficiency on root systems and leaf surfaces (Taylor et al., 2022). Correspondingly, our study further demonstrates that exogenous K^+^ significantly enhances the colonization ability of *P. protegens* SN15-2 on leaves. Collectively, these findings indicate that, in addition to improving desiccation tolerance, K^+^ can enhance the biocontrol efficacy of *P. protegens* by promoting its adhesion and colonization abilities.

Improving the survival rate during the preparation of bacterial formulations conveys significant benefits for their production processes and commercial applications. Bacterial cell encapsulation has been widely used in agriculture to create protective structures that enable immobilization, protection, and controlled release (Saberi Riseh et al., 2025). Sodium alginate is commonly used for encapsulating Pseudomonas species (Pour et al., 2019). Based on the findings that K^+^ enhance the desiccation tolerance of *P. protegens*, this study further demonstrates that K^+^ increase the survival of *P. protegens* encapsulated in sodium alginate microcapsules during processing and storage. These results provide a simple and effective protective strategy for preparing *P. protegens* formulations, thereby facilitating its large-scale application. Additionally, it was confirmed that overexpression of *gmd* improves the desiccation stress tolerance of *P. protegens*, offering valuable insights for constructing engineered strains with enhanced stress resistance.

The transition of this exogenous K^+^ strategy from laboratory to practice appears highly feasible. Potassium salts such as KCl are low-cost, widely available, agriculturally compatible, presenting minimal barriers to integration into large-scale fermentation and formulation processes. This approach represents a simple, cost-effective step that could be readily incorporated before cell drying to significantly improve the viability of *P. protegens* in biocontrol products. Beyond production, a critical question is whether the protective and fitness benefits conferred by K^+^ extend to the complex soil and rhizosphere environment where the bacterium must ultimately function. The improved adhesion and colonization traits induced by K^+^ are likely to enhance root and soil colonization in the field. However, soil is a dynamic and heterogeneous matrix where introduced bacteria face multifaceted abiotic and biotic stresses. To confirm the full practical value of this strategy, future validation through pot and field trials will be essential, directly comparing the persistence and efficacy of K^+^-treated versus standard *P. protegens* inoculants in diverse soils. In summary, this study demonstrates that K^+^ significantly enhance the desiccation tolerance, adhesion, and colonization capacity of *P. protegens*, while providing initial insights into the underlying mechanisms. These findings offer a practical and promising strategy for advancing the development and application of *P. protegens* biocontrol agents.

## Data Availability

The transcriptome data for this study are publicly available in the Sequence Read Archive via the accession number PRJNA1391811.
