# Supplementary material for: Exogenous K+ enhances the desiccation tolerance and adhesion of Pseudomonas protegens SN15-2
Source: Front Microbiol. 2026 Mar 16;17:1774231. doi: 10.3389/fmicb.2026.1774231 (PMC13033688; doi:10.3389/fmicb.2026.1774231)
Supplement: Supplementary file 1 [file Data_Sheet_1.docx]

**Exogenous K⁺ Enhances the Desiccation Tolerance and Adhesion of *Pseudomonas protegens* SN15-2**


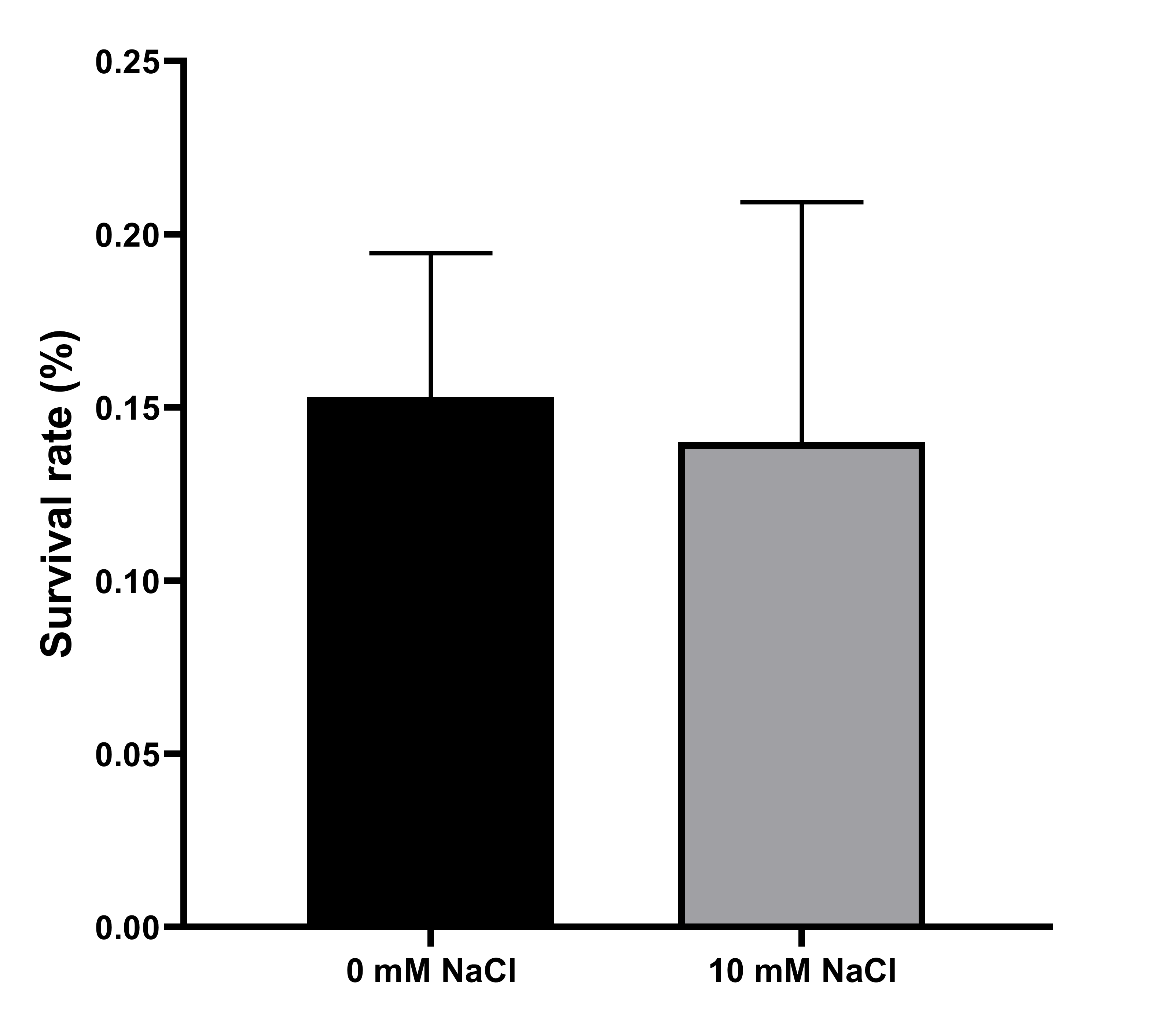


**Fig. S1**. Effect of NaCl supplementation on the desiccation tolerance of *P. protegens* SN15-2


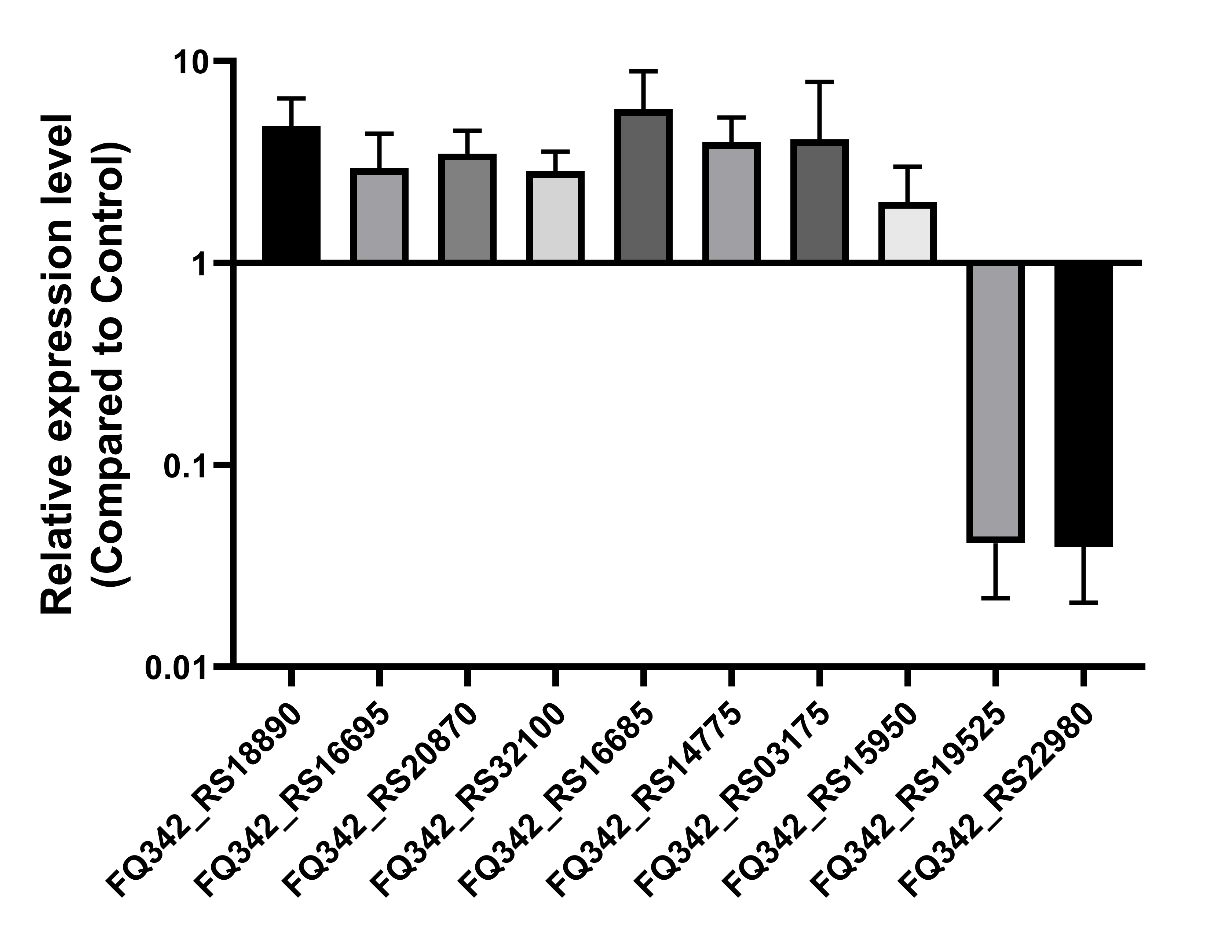


**Fig. S2.**  qRT-PCR analysis of the transcription levels of 10 genes selected from RNA-seq data. Error bars indicate the standard deviations (n=3).

**Table S1.** Oligonucleotides used in this study

| Primer name | Nucleotide sequence (5'-3') | | PCR product | |
| --- | --- | --- | --- | --- |
| **Primers for plasmid construction** | |  |  | |
| *gmd-E*1 | ATCCCCCGGGCTGCAGGAATTCtcacgtacctacacttcctatg | | complete ORF of *gmd* used to generate pBBR-*gmd* | |
| *gmd-E2* | GGTCGACGGTATCGATAAGCTTtatgaaacgcgcagttatcac | | complete ORF of *gmd* used to generate pBBR-*gmd* | |
| **Primer for RT-qPCR assay** |  | |  | |
| FQ342_RS18890-1 | TCGTTGACCGCTCGATTGAA | |  | |
| FQ342_RS18890-2 | TGACGACACCCCAGATTGTG | |  | |
| FQ342_RS16695-1 | TGTTTTCCTTGGTTGCTGCG | |  | |
| FQ342_RS16695-2 | GGCCACAAATCCTGTACCCA | |  | |
| FQ342_RS20870-1 | GCTCTCATACCGTGCCTACC | |  | |
| FQ342_RS20870-2 | TGCACCTGCTCCCCATAAAG | |  | |
| FQ342_RS32100-1 | GATGGCTGGCGTCTAGTGTT | |  | |
| FQ342_RS32100-2 | AAACTGCTCTACACGCTCCC | |  | |
| FQ342_RS16685-1 | TTGGTCATACGGTGTTGCGA | |  | |
| FQ342_RS16685-2 | ACTCGCAGGATGGTACTTGC | |  | |
| FQ342_RS14775-1 | CGTCGGTCGAGTTCTGTGAA | |  | |
| FQ342_RS14775-2 | GACGAAACTCTGAGCCGCTA | |  | |
| FQ342_RS03175-1 | CGTTGGCGCCTATGACAATG | |  | |
| FQ342_RS03175-2 | GTGGCCTTGACGTCCTTGTA | |  | |
| FQ342_RS15950-1 | GACTGCGACCTGTCGATCAT | |  | |
| FQ342_RS15950-2 | AACGGTAGCCGAGCCTTTAC | |  | |
| FQ342_RS19525-1 | GTTCCTGGTGGGCATGATGA | |  | |
| FQ342_RS19525-2 | GGTGAAACCGAGGATGCAGA | |  | |
| FQ342_RS22980-1 | GTGGTTCAACAACGCCAAGG | |  | |
| FQ342_RS22980-2 | CTGTCCGGCTTTCAAGGTCT | |  | |
| 16S-1 | CTACGGCTACCTTGTTAC | |  |  |
| 16S-2 | GATGGATTGGTGCCTTCG | |  |  |
